# Beyond awareness: Defining AI readiness in Emergency Medicine across health systems

**DOI:** 10.1016/j.afjem.2026.101017

**Published:** 2026-09-16

**Authors:** Mert Gültekin

**Affiliations:** Department of Emergency Medicine, Şanlıurfa Training and Research Hospital, Şanlıurfa, 63200, Türkiye

## Abstract

• Frames AI readiness as more than awareness or enthusiasm in emergency care.
• Highlights governance, validation, infrastructure, and workflow integration.
• Uses evidence from Türkiye to contextualise challenges reported in African EDs.
• Supports context-sensitive AI implementation in resource-variable African settings.

**Dear Editor,**

Tadesse et al. [1] provide timely multi-country evidence on how Emergency Medicine clinicians in selected sub-Saharan African settings perceive artificial intelligence (AI). Their most informative finding may be the gap between individual exposure and institutional preparation: 88.2% reported a basic understanding of AI and 73.0% had used AI tools, yet only 14.2% had received formal training and 12.0% reported an institutional AI implementation strategy [1]. Stassen [2] aptly described this pattern as "AI-aware but not AI-ready". I suggest that the next question is how readiness should be defined, and whether this gap is unique to resource-constrained settings or reflects a broader challenge in emergency medicine.

Evidence from Türkiye offers a useful, although not directly comparable, perspective. In a national survey of 167 emergency medicine specialists, Ahun et al. [3] found that 54.5% considered AI-assisted pandemic triage beneficial for patients and 70.06% considered it beneficial for clinicians; 63.47% supported shared responsibility between clinicians and AI developers or manufacturers for complications. In a separate cross-sectional evaluation involving 745 emergency department patients, Colakca et al. [4] reported an overall triage accuracy of 76.6% for ChatGPT compared with an expert committee, with stronger performance for high-acuity categories (94.9% accuracy; kappa = 0.828).

These Turkish studies should not be numerically contrasted with the survey by Tadesse et al., because they assess different populations, technologies, outcomes, and periods. Nevertheless, together they illustrate an important distinction: favourable clinician attitudes and promising technical performance do not, by themselves, establish health-system readiness. A systematic review similarly found that emergency department AI research has concentrated on triage and clinical decision support while real-world implementation remains relatively nascent [5]

I therefore propose viewing AI readiness as a multidimensional construct encompassing clinician literacy and formal training, locally validated performance, institutional governance and accountability, secure digital infrastructure, and workflow integration with meaningful human oversight. Future multi-country surveys could measure these domains separately using common operational definitions rather than relying primarily on awareness or self-reported use.

This framework may make cross-system comparisons more informative and less dependent on resource labels alone. Tadesse et al. have provided valuable data from an underrepresented region; their findings also prompt a broader question for emergency medicine: are we measuring AI use, or the capacity to use AI safely and sustainably?

## Funding

This work received no specific grant from any funding agency in the public, commercial, or not-for-profit sectors.

## Declaration of generative AI and AI-assisted technologies in the writing process

During the preparation of this work, the author used ChatGPT (OpenAI) to assist with drafting, language refinement, and organisation of the manuscript. The author subsequently reviewed and edited the content and takes full responsibility for the content of the publication.

## CRediT authorship contribution statement

**Mert Gültekin:** Conceptualization, Investigation, Supervision, Writing – original draft, Writing – review & editing.

## Declaration of competing interest

The author declared no conflicts of interest.
